# Extracorporeal life support as bridge to lung transplantation: a systematic review

**DOI:** 10.1186/s13054-014-0686-7

**Published:** 2015-01-22

**Authors:** Davide Chiumello, Silvia Coppola, Sara Froio, Andrea Colombo, Lorenzo Del Sorbo

**Affiliations:** Dipartimento di Anestesia, Rianimazione (Intensiva e Subintensiva) e Terapia del Dolore, Fondazione IRCCS Ca’ Granda, Ospedale Maggiore Policlinico, Via F. Sforza 35, 20122 Milano, Italy; Dipartimento di Fisiopatologia Medico-Chirurgica e dei Trapianti, Università degli Studi di Milano, Milano, Italy; Interdepartmental Division of Critical Care Medicine, University of Toronto, Toronto, Canada; Dipartimento di Anestesiologia e Medicina degli Stati Critici, Università di Torino, Torino, Italy

## Abstract

**Introduction:**

Patients with acute respiratory failure requiring respiratory support with invasive mechanical ventilation while awaiting lung transplantation are at a high risk of death. Extracorporeal membrane oxygenation (ECMO) has been proposed as an alternative bridging strategy to mechanical ventilation. The aim of this study was to assess the current evidence regarding how the ECMO bridge influences patients’ survival and length of hospital stay.

**Methods:**

We performed a systematic review by searching PubMed, EMBASE and the bibliographies of retrieved articles. Three reviewers independently screened citation titles and abstracts and agreement was reached by consensus. We selected studies enrolling patients who received ECMO with the intention to bridge lung transplant. We included randomized controlled trials (RCTs), case–control studies and case series with ten or more patients. Outcomes of interest included survival and length of hospital stay. Quantitative data summaries were made when feasible.

**Results:**

We identified 82 studies, of which 14 were included in the final analysis. All 14 were retrospective studies which enrolled 441 patients in total. Because of the broad heterogeneity among the studies we did not perform a meta-analysis. The mortality rate of patients on ECMO before lung transplant and the one-year survival ranged from 10% to 50% and 50% to 90%, respectively. The intensive care and hospital length of stay ranged between a median of 15 to 47 days and 22 to 47 days, respectively. There was a general paucity of high-quality data and significant heterogeneity among studies in the enrolled patients and technology used, which confounded analysis.

**Conclusions:**

In most of the studies, patients on ECMO while awaiting lung transplantation also received invasive mechanical ventilation. Therefore, whether ECMO as an alternative, rather than an adjunction, to invasive mechanical ventilation is a better bridging strategy to lung transplantation still remains an unresolved issue. ECMO support as a bridge for these patients could provide acceptable one-year survival. Future studies are needed to investigate ECMO as part of an algorithm of care for patients with end-stage lung disease.

## Introduction

Lung transplant is considered an established treatment for patients with end-stage chronic respiratory failure [1]. Since the first successful report in 1983 by Cooper and colleagues [2], more than 30,000 lung transplants have been done worldwide [3]. The significantly larger number of candidates than available organs explains the long waiting times and high risk of perioperative morbidity and mortality [1,4–6]. Contributing to this high mortality rate is also the lack of efficacious and safe means of artificial respiratory support for patients awaiting the transplant once they develop acute respiratory failure with refractory hypoxemia and hypercapnia [4,7]. Mechanical ventilation *per se* can aggravate acute respiratory failure and hemodynamic instability, increasing the risk of ventilator-associated pneumonia and ventilator-induced lung injury [7–10]. Mechanically ventilated pre-transplant patients have been reported to have significantly higher post-transplant mortality rates than non-ventilated patients [11,12].

More than three decades ago, extracorporeal membrane oxygenation (ECMO) was introduced to manage patients on the lung transplant waiting list who were dying of acute respiratory failure refractory to mechanical ventilation [4,13]. The first report of ECMO as a feasible bridging strategy to transplantation goes back to 1975: the patient survived transplantation but died shortly after from complications of infection [14]. After a randomized trial that suggested that ECMO was associated with a worse outcome than mechanical ventilation in patients with acute respiratory failure [15], ECMO as a bridge to lung transplant fell into disuse [4].

More recently, however, thanks to improvements in technology, safety profile and manageability of extracorporeal life support strategies [16,17], ECMO has been reintroduced in some centers as an option for patients with severe respiratory failure awaiting lung transplant [4,10,13,18–22]. The number of lung transplant candidates who could benefit from ECMO is now significantly larger [13,19,20,22]. A report by the United Network of Organ Sharing (UNOS) showed that, despite its complexity and side effects, the use of ECMO as a bridge to lung transplant has risen by 150% in the two last years compared to the previous decades (1970 to 2010) [4]. However, given the small numbers of transplantable lungs, bridging lung-transplant candidates on ECMO has raised ethical concerns about the risk of potentially selecting for transplantation, very severely ill patients with the risk of poor post-transplant outcome. Instead, according to the lung allocation score (LAS) system, organs should be allocated to patients who have the greatest need, such as those on ECMO, but who are also likely to benefit most from the transplant [5,6,23,24].

In this perspective, new advances have demonstrated the potential of ECMO as an alternative to mechanical ventilation in awake, spontaneously breathing patients. With awake-ECMO patients preserve their muscle tone, with greater possibility of early mobilization and participation in intensive physical therapy, thus improving their condition before a lung transplant and making for a better post-transplant outcome [20,25–29].

Given the lack of definitive data on the efficacy of ECMO as bridge to lung transplantation, and the consequent lack of a clear consensus on this strategy [6], we conducted a systematic review to assess the current evidence on the use of ECMO in patients with advanced respiratory failure awaiting lung transplant.

## Materials and methods

### Search strategy and selection of studies

Our search used the statement - preferred reporting items for systematic reviews and meta-analyses (PRISMA) - as a guide [30]. We made a computerized search of MEDLINE/PubMED and EMBASE databases from January 2000 to June 2014. Our search was limited to studies on humans and adults. We limited the selection to studies written in English, French, German or Spanish. We used the following search keywords and terms: “preoperative extracorporeal membrane oxygenation” OR “preoperative ECMO” AND “lung transplantation”, “extracorporeal membrane oxygenation” OR “ECMO” AND “bridge to lung transplantation”, “ambulatory extracorporeal membrane oxygenation” OR “ambulatory ECMO” AND “lung transplantation”.

Three reviewers (DC, SC and SF) independently screened citation titles and abstracts. We looked through the references of all articles retrieved and reviewed the articles to identify additional potentially eligible studies. In case of disagreement the authors reviewed the article in question together until they reached a consensus. We deleted duplicate papers. All potentially eligible papers were retrieved in full and assessed to confirm eligibility.

We screened studies for relevance that enrolled patients awaiting lung transplant who were admitted to ICU to receive ECMO support as a bridging procedure, including veno-venous approach and veno-arterial support. We excluded studies that enrolled patients treated with pump-free extracorporeal interventional lung-assist devices. We included studies enrolling at least ten patients on ECMO bridging. Data were abstracted in duplicate by two reviewers (SC and SF) and any discrepancies were solved by discussion.

The following information was collected in a datasheet: publication (first author’s name, year, journal), study design, number of enrolled patients on ECMO bridge, number of patients who died while awaiting transplant, type of ECMO support, timing of ECMO bridge, outcomes, survival after lung transplant.

### Assessment of methodological quality

Two authors (SC and SF) independently assessed the methodological quality of the studies. They employed critical appraisal skills program (CASP) tools using the CASP checklist for case-control studies [31].

## Results

### Study selection and characteristics

**Figure 1:**
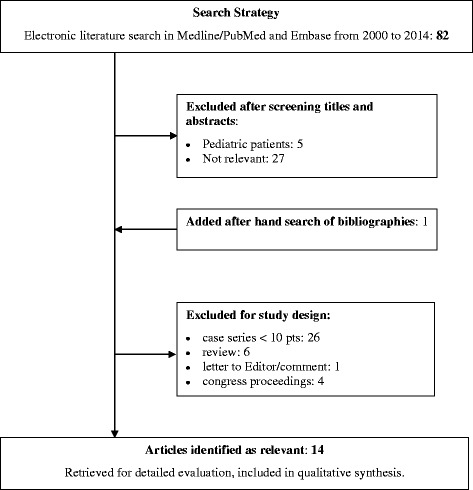
**Flow chart of the study selection process.** Pts, patients.

The initial search strategy identified 82 potentially eligible studies (Figure 1); 69 studies were excluded for the following reasons: 5 involved pediatric patients, 27 were deemed not relevant and 37 were case series, reviews, letters or congress proceedings. After a hand search of the bibliographies, 14 articles met the inclusion criteria and were considered for this systematic review. All were retrospective studies.

The main characteristics of patients enrolled are summarized in Table 1. The studies were published in the three years from 2010 to 2013, with 441 patients enrolled from 1987 to 2012. Eight studies were done in the United States [10,11,20,26,32–34], three in Italy [35–37] and one each in Sweden, Germany and France [38–40]. Ten studies reported a severity score before ECMO bridge: six reported the LAS [10,11,20,32,33,41] and four the SOFA score [26,35,37,39].

**Table 1:** **Characteristics of patients who underwent ECMO bridge to lung transplant and were enrolled in the selected studies**

**Author, year**	**Patients, number**	**Age, years**	**Sex male, n (%)**	**Diagnosis**	**Ventilation strategy**	**Bridge time, days**	**Severity score pre-bridge**
Mason, 2010 [11]	51	39 ± 22	25 (49%)	PF 27%; COPD 19%; CF 12%; PH 9.8%; sarcoidosis 2%; other 20%	na	na	LAS 54 ± 21
Bermudez, 2011 [34]	17	40 ± 14	7 (41%)	PF 35%; Re-LTx 35%; CF 23%; COPD 6%	MV	3.2 (0 to 49)	na
Hammainen, 2011 [38]	16	41 ± 8^a^	7 (58%)^a^	PF 37%^a^; PH 15%^a^; CF 8%^a^; ARDS 8%^a^; IP 8%^a^; PVOD 8%^a^; BOS 8%^a^; PGD 8%^a^	na	12 (1 to 59)	na
Shafii, 2012 [41]	19	44 (23 to 60)	10 (53%)	IP 68%; CF 16%; PH 16%	MV 13	6 ± 5	LAS 87 (64 to 95)
Nosotti, 2012	11	34 ± 13	5 (45%)	na	Awake 7 MV 4	12.1 ± 14.7	SOFA 4.9 ± 1.4
Javidfar, 2012 [20]	18	34 (22 to 50)	8 (45%)	CF 44%; PF 33%; PH 11%; Other 11%	Awake 6	11.5 (6 to 18)	LAS 93 (90 to 94)
George, 2012 [10]	122	48 ± 16	74 (60%)	PF 29.5%; CF 11.5%; COPD 10.7%; PH 2.5%; other 45,8%	na	na	LAS 73.9 ± 21.4
Fuehner, 2012 [26]	26	44 (23 to 62)	21 (81%)	PF 35%; PH 27%; CF 19%; BOS 12%; sarcoidosis 4%	Awake 19 MV 7	9 (1 to 45)	SOFA 7 (6 to 12)
Hoopes, 2013 [32]	31	45 ± 15	21 (67%)	PF 29%; CF 23%; ILD 13%; ARDS 10%; PVOD 10%; PH 6%; BOS 3%; IP 3%; CWP 3%	Ambulatory 18 13 VM	11 (2 to 53)	LAS >50
Anile, 2013 [36]	12	na	na	CF 92%; histiocytosis 8%	Awake 2 MV 10	6 ± 2.1	na
Toyoda, 2013 [33]	31	46 ± 15^a^	10 (42%)^a^	PF 33%^a^; CF 21%^a^; Re-LTx 13%^a^; scleroderma 13%^a^; bronchiectasis 8%^a^; COPD 4%^a^; sarcoidosis 4%^a^; PH 4%^a^	MV^a^	7.1 ± 10	LAS 87 ± 9^a^
Weig, 2013 [39]	26	36 (30 to 51)^a^	14 (54%)	PF 62%; CF 23%; COPD 4%; Re-LTx 4%; Lung cancer 4%; sarcoidosis 4%	na	16 (8.8 to 25)^a^	SOFA 9 (8.5 to 10.5)^a^
Crotti, 2013 [35]	25	41 ± 12	na	PF 52%; CF 16%; PH 16%; Re-LTx 12%; ARDS 4%	Awake 10 MV 15	5.8 ± 4.5 versus 29.8 ± 11.5^b^	SOFA 5.6 ± 1.9
Lafarge, 2013 [40]	36	31 (22 to 48)	19 (53%)	CF 56%; PF 30%; other 14%	MV	3.5 (2 to 7)	na

Data presented in this table refer to patients underwent ECMO support with the intention to bridge to lung transplantation. ^a^Transplanted patients (when data for all enrolled patients are not available; Hammainen *et al*., all data; Toyoda, all data; Weig *et al*., age, ECMO bridge time and SOFA; Anile, diagnosis). ECMO bridge time (days) and the pre-bridge severity score are expressed as mean ± standard deviation or median and range. When no descriptive cumulative data for the overall population are provided, they are calculated from raw data presented in the original papers. ^b^Data refer to patients divided according to waiting time on ECMO: up to 14 days or longer. Pts, patients; ECMO, extracorporeal membrane oxygenation; PF, pulmonary fibrosis; COPD, chronic obstructive pulmonary disease; CF, cystic fibrosis; PH, Pulmonary hypertension; Re-LTx, Re-lung transplantation; ARDS, acute respiratory distress syndrome; IP, interstitial pneumonia; PVOD, pulmonary veno-occlusive disease; BOS, bronchiolitis obliterans syndrome; PGD, primary graft dysfunction; ILD, interstitial lung disease; CWP, coal workers pneumoconiosis; MV, mechanical ventilation; LAS, lung allocation score; SOFA, sequential organ failure assessment; na, not available.

In all the studies, depending on the clinical conditions, either a veno-venous or veno-arterial ECMO was used [10,11,20,26,32–41]. The strategy during ECMO bridging was invasive mechanical ventilation in four studies [33,34,40,41], and spontaneous breathing or invasive ventilation according to clinical needs in six studies [20,26,32,35–37]. Unfortunately, four studies provided no information about the ventilation strategy during ECMO bridging [10,11,38,39].

The time on ECMO bridging before lung transplantation ranged from a median of 3.2 days [34] to 16 days [39] (Table 1). Crotti *et al*., dividing patients according to whether the waiting time on ECMO was up to 14 days or longer, observed an ECMO bridging duration of 29.8 ± 11.5 days in patients who received a transplant after waiting more than 14 days on ECMO [35].

### Quality of studies

All 14 studies included were retrospective analyses. The sample size ranged from 11 [37] to 122 [10]. Eight studies were single-center trials. Seven had no control group [20,35–40]. Only six studies used the LAS system to describe the severity of their pre-bridge population [10,11,20,32,33,41]. Across all 14 studies there were substantial differences in the inclusion criteria for patients, ECMO program times, and ECMO support technologies including VV and VA. Because of this, we cannot exclude a possible confounding role of some important factors such as diagnosis and comorbidity at the beginning of the bridge. Four studies [10,11,32,37] only examined patients who were successfully bridged to transplantation without mentioning patients who had died while on the waiting list.

Post-LTx complications differed among studies and were therefore not comparable. For example, six studies did not report the incidence of primary graft dysfunction. Over half presented no data on the need for post-LTx mechanical ventilation (Table 2).

**Table 2:** **Outcomes**

**Author, year**	**Ltx/total patients, n**	**Died before Ltx, n (%)**	**Type of bypass**	**Survival at 1 yr post-LTx, %**	**Length of stay**	**MV, days**
					**post-LTx, days**	**post-LTx**
Mason, 2010 [11]	51/51	na	na	50%	24 (9 to 55) H	na
Bermudez, 2011 [34]	14/17	3 (17%): neurologic dysfunction, thrombosis	VV, VA	74%	16 (3 to 40) ICU	12 (2 to 20)
Hammainen, 2011 [38]	13/16	3 (19%): septic MOF	VV, VA	92%	22 (3 to 63) ICU	na
Shafii, 2012 [41]	14/19	5 (26%): septic MOF 2, DIC 2, anoxic brain injury 1	VV, VA	75%	42 (19 to 175) H	22 (5 to 125)
					15 (8 to 42) ICU	
Nosotti, 2012	11/11	na	VV	87% and 50%^b^	47.6 ± 21.9 H	27.1 ± 20.7
					30 ± 20.4 ICU	
Javidfar, 2012 [20]	10/18^a^	8 (44%): pneumonia 1, MOF 6, CA 1	VV,VA	60%	22 (18 to 33) H	na
					47 (41 to 52) ICU	
George, 2012 [10]	122/122	na	na	57.6%	32 (16.5-60) H	na
Fuehner, 2012 [26]	20/26	6 (23%): CA 2, septic MOF 4	VV,VA	6-month 80%	38 (20 to 87) H	14 (0 to 64)
					18 (1 to 69) ICU	
Hoopes, 2013 [32]	31/31	na	VA, VV	93%	31 (12 to 86)^e^ H	na
Anile, 2013 [36]	7/12	5 (41%)	VV, VA	85.7%	29 (15 to 59) H	<5
Toyoda, 2013 [33]	24/31	7 (22%)	VV,VA	74%	46 median H	na
Weig, 2013 [39]	13/26	13 (50%): acute liver failure 7, thoracic bleeding 3, cerebral hemorrhage 1,PE 2	VV,VA	54%	na	na
Crotti, 2013 [35]	17/25	8 (32%): MOF 3, septic shock 2, cardiogenic shock 2, intestinal ischemia 1	VV,VA	82% and 29%^c^	na	12.2 ± 11.9^d^
						45.3 ± 33.5
Lafarge, 2013 [40]	30/36	6 (17%): GI bleeding 1, DIC 1, cerebral hemorrhage 1, CA 1, septic shock 1, therapeutic limitation 1	VV,VA,CPB	66.5%	na	na

Data are expressed as mean ± standard deviation or median and range. Mason *et al*., Nosotti *et al*., Hoopes *et al*. and George *et al*. enrolled transplanted patients. ^a^Three of the eight patients who died had transiently recovered their baseline function and were weaned from ECMO support; they subsequently died before LTx. ^b^ECMO group: 87% awake (7 pts); mechanical ventilation ECMO group: 50% (4 pts); ^c^82% patients on ECMO bridge <14 days (early): 29% patients on ECMO bridge >14 days (late); ^d^12.2 ± 11.9 days (early group) −45.3 ± 33.5 (late group). ^e^Mean (range). LTx, lung transplant; CA, cardiac arrest; MOF, multi-organ failure; DIC, disseminated intravascular coagulation; GI, gastrointestinal; VV, veno-venous; VA, veno-arterial; CPB, cardiopulmonary by-pass; MV, mechanical ventilation; LOS, length of stay; H, hospital; na, not available.

In addition, a learning-curve bias effect cannot be formally excluded in studies enrolling patients over a long period. Regional differences among studies in organ allocation policy, institutional differences in the logistic design and deployment criteria of extracorporeal circuits and surgeon-specific preferences about organ selection and operative technique also pose limitations for a reliable comparison. Given the substantial heterogeneity across studies we did not attempt a meta-analysis because it would not have yielded clinically meaningful results; data were descriptively summarized.

### Survival

The mortality rate of patients on ECMO before lung transplant was reported in ten studies and ranged between 17% and 50% with multiple organ failure, septic shock, cardiac failure and bleeding described as the most frequent causes (Table 2). Interestingly, in the study by Weig *et al*. liver failure developed in up to half the patients who died while awaiting lung transplant [39].

All 14 studies reported the post-transplant one-year survival rates. In five studies it ranged from 50% to 70% [10,11,20,39,40], in four 70% to 90% [33,34,36,41] and in two up to 90% [32,38] (Table 2). When patients were stratified according to the ventilation strategy during ECMO bridge [37] or according to the ECMO bridge duration [35] one-year survival was significantly better in spontaneously breathing patients than mechanically ventilated ones (85% versus 50%) or when the ECMO bridge duration was shorter than 14 days (82% versus 29%). Fuehner *et al*. reported only a six-month survival rate of 80%, for 19 patients on spontaneous breathing and 7 patients on mechanical ventilation [26]. Similarly, Hoopes *et al*. found high one-year survival in 26 ECMO bridge transplanted patients, 18 of whom were ambulatory at transplantation [32].

Bermudez *et al*. found the survival rate in patients who received ECMO support was similar to a control group that was mechanically ventilated before transplant (74% versus 78%) [34]. However, they did not report the LAS score comparing the clinical status of the two groups. Nonetheless, in patients with similar LAS scores (54 ± 22 and 54 ± 21) Mason *et al*. also reported similar survival rates for those bridged with ECMO and those with mechanical ventilation (50% and 62%). However, this was significantly lower than for unsupported patients (79%) although this group also had lower LAS scores (40 ± 11) [11].

Despite significantly higher LAS scores (87 ± 9 versus 44 ± 15) Toyoda *et al*. found comparable survival rates (74% and 83%) in the ECMO group and in patients who received the transplant without bridge support [33]. Hoopes *et al*. reported a post-transplant survival rate of ECMO patients comparable to or slightly higher than that of patients with a LAS score higher than 50 transplanted without bridge support, from the UNOS database [32]. In contrast, George *et al*. noted significantly lower survival in ECMO patients and in mechanically ventilated patients than in unsupported patients with the highest LAS quartile (58%, 68% and 81%) [10]. However, survival of ECMO patients rose significantly, from 30% in 2005 to 75%, in 2010 [10].

Fuehner *et al*., applying an awake-ECMO strategy avoiding intubation and general anesthesia, found significantly better survival than with mechanical ventilation (80% versus 50%) [26]. Similarly, Crotti *et al*. reported a survival rate of 75% in unsupported ECMO patients [35]. Although these two studies suggest that ECMO support as an alternative to invasive mechanical ventilation seems to achieve better outcomes than when combined with invasive respiratory assistance, these data come from very experienced centers and may not be generalizable. Whether ECMO alone is a better bridging strategy to lung transplant than invasive mechanical ventilation has yet to be investigated more systematically.

George *et al*., stratifying the data by age and by diagnosis, found that ECMO-bridged patients younger than 62 years had better one-year survival than older patients (65% versus 38%) as did patients with chronic obstructive pulmonary disease, while patients bridged to re-lung transplant had the worst outcomes (84% vs 40%) [10].

### Length of stay and ECMO-related complications

The ICU and hospital lengths of stay were reported in six [20,26,34,37,38,41] and nine studies [10,11,20,26,32,33,36,37,41] respectively and the medians ranged from 15 to 47 days [20,34] and 22 to 47 days [20] (Table 2). With regard to the ventilation strategy Crotti *et al*. found that non-invasive ventilation during ECMO bridge was associated with significantly shorter ICU and hospital stays than invasive mechanical ventilation (31 ± 19 versus 84 ± 44 days and 52 ± 28 versus 119 ± 55 days respectively [35]. Similarly, Nosotti *et al.* found a shorter mean ICU stay after lung transplantation in the awake-ECMO group than the mechanically ventilated ECMO group, but the difference was not statistically significant [37].

Postoperative complications in transplanted patients before discharge from hospital are described in Table 3. The most frequent were the need for tracheostomy (up to 77%) [38], pulmonary graft dysfunction requiring post-lung transplant ECMO (54%) [33], pneumonia (52%) [34], kidney failure treated with renal replacement therapy and critical illness polyneuropathy/myopathy (up to 70%) [35]. The most frequent causes of death after lung transplantation in ECMO-bridged patients before discharge were sepsis, multiple organ failure, bleeding and primary graft dysfunction.

**Table 3:** **Complications in patients discharged alive from hospital and causes of death in hospital after lung transplant**

**Complications in patients discharged alive from hospital**	
▪	Respiratory complications:
	• PGD requiring Post-Ltx ECMO 47%^[34]^; 21%^[41]a^; 20%^[20]a^; 54%^[33]a^
	• PGD 72 hours 3^rd^ grade 36%^[37]^; 15%^[39]a^; 35%^[35]a^
	• Tracheostomy 41%^[34]^; 77%^[38]a^; 64%^[41]a^; 27%^[26]^
	• Bronchopleura fistula 8%^[38]a^; 14%^[36]a^
	• Open chest management 50%^[41]a^; 8%^[39]a^
	• Acute rejection 15%^[38]a^; 28%^[36]a^
▪	Acute kidney injury 35%^[34]^; 12%^[35]a^
▪	Renal replacement therapy 23%^[34]^; 38%^[38]a^; 43%^[41]a^; 4%^[26]^; 14%^[36]a^; 54%^[37]^; 12%^[35]^
▪	Infective complications:
	• Pneumonia 52%^[34]^
	• Sepsis 23%^[34]^; 19%^[26]^; 14%^[36]a^
▪	Hemorrhagic complications:
	• GI bleeding 5%^[34]^
	• Bleeding from femoral artery 5%^[34]^
	• Re-op. for bleeding 15%^[38]a^; 29%^[41]a^; 36%^[37]^; 28%^[36]a^; 15%^[39]a^
	• Hemorrhage 31%^[26]^; 35%^[35]a^
	• Massive hemoptysis 15%^[26]^
▪	Neurological complications:
	• Cerebral hemorrhage 5%^[34]^
	• Stroke 8%^[38]a^
	• Ischemia thoracic spinal cord 3%^[32]^
	• CIP/CIM 31%^[38]a^; 64%^[37]^; 70%^[35]a^
▪	Digital ischemia 17%^[34]^; 14%^[36]a^
**Causes of death in hospital after LTx**	
▪	MOF 6%^[34]^; 15%^[26]^; 7%^[40]a^
▪	Sepsis 18%^[34]^; 14%^[41]a^; 10%^[40]a^
▪	Acute colonic rupture 8%^[38]a^
▪	Cardiogenic shock 6%^[34]^
▪	Cardiac arrest 10%^[40]a^
▪	Bleeding 9%^[37]^; 31%^[39]a^; 3%^[40]a^
▪	Neurological event 3%^[32]^
▪	Lung cancer 5%^[26]^
▪	Bronchopleural fistula 3%^[40]a^
▪	PGD 31%^[39]a^
▪	Open chest treatment 23%^[39]a^
▪	Other 18%^[35]a^

^a^ percentages of transplanted patients: Shafii *et al*., Hammainen *et al*., Javidfar *et al*., Toyoda *et al*., Crotti *et al*., Anile *et al*., Lafarge *et al*., Weig *et al*.. When the percentages of complications were not available, complications referring to the overall enrolled population are reported.

ECMO, Extracorporeal membrane oxygenation; Post-LTx, Post-lung transplantation; PGD, Primary graft dysfunction; GI, Gastrointestinal; MOF, Multi-organ failure; Re-op., Re-operation; CIP/CIM, Critical illness polyneuropathy/critical illness myopathy.

## Discussion

This systematic review suggests that the use of ECMO support as a bridge strategy for patients awaiting lung transplant is associated with high perioperative morbidity and mortality but achieves acceptable one-year survival, very similar to that of mechanically ventilated patients. The initial goal of this systematic review was to verify the feasibility of meta-analysis of the retrieved data. However, in view of the wide heterogeneity of the selected studies we decided to make only a qualitative summary of the selected literature. The 14 studies included are not randomized trials, but retrospective analyses of case series in which the selection of patients and ECMO treatment were discretional to the center and not homogeneous, thus possibly affecting the efficacy of the treatment and certainly preventing the results being evaluated in a meta-analysis.

Lung transplantation is the only option for patients with end-stage lung failure. However, organ supply is grossly inadequate compared to the large numbers of patients awaiting transplant, so mortality in the waiting list is still very high [11]. In this setting, mechanical ventilation and ECMO are the only supportive strategies available to prolong these patients’ lives, increasing their chances of receiving suitable organs.

Although the main benefit of mechanical ventilation is to improve gas exchange [12], it can in fact open the way to pulmonary infection, sepsis and muscle atrophy, prolonging weaning after lung transplant and making it difficult [11,42,43]. ECMO, on the other hand, could potentially provide adequate respiratory and hemodynamic support, with fewer of the side effects of mechanical ventilation in patients awaiting lung transplant, offering an alternative bridging strategy [7,13,19,20,22,44]. However, many transplant centers still consider ECMO a contraindication to lung transplant given the mixed outcomes in patients transplanted from ECMO [6]. This systematic review found that ECMO-bridged patients had satisfactory post-transplant survival, similar to patients bridged with mechanical ventilation. ECMO has helped to save numerous high-risk transplant candidates with otherwise acutely lethal conditions.

Proper patient selection for ECMO is clearly essential for a good long-term outcome. In the studies reviewed here, ECMO was not considered suitable for patients with sepsis, neurologic impairment, profound malnutrition [41] or severe graft dysfunction after lung transplantation [33], whereas advanced age (>50 years) was not a contraindication [41]. The clinical conditions of patients supported with pre-transplant ECMO are usually more critical than those of the population awaiting lung transplant and this may have a negative influence on their overall outcome [16]. However, this systematic review found that at least in selected reports the post-transplant outcome of ECMO-bridged patients was comparable to recipients who did not receive pre-transplant support. Any excessive reduction of the post-transplant survival rate of ECMO-bridged recipients would obviously defeat the principle of allocating transplantable lungs on the basis not only of the severity of their clinical condition, but also the potential long-term benefit. The definition of clinical parameters predicting survival for ECMO-bridged patients would certainly help clarify this problematic question [22,45].

In most of the studies reviewed patients bridged with ECMO were also supported with mechanical ventilation, thus combining two invasive means of respiratory support, each with potentially harmful side effects. Recent reports do suggest that invasive mechanical ventilation may still be considered an effective bridge. Mason *et al.* showed that the unadjusted post-transplant survival at one year was 62% for recipients bridged with mechanical ventilation, 50% for those bridged with ECMO, and 79% for unsupported patients [11]. Vermeijden *et al.* compared the outcomes of 13 lung transplant recipients bridged with invasive mechanical ventilation with 70 controls who received no pre-transplant support [46]. Interestingly, the two groups had similar post-transplant survival and incidence of primary graft dysfunction.

To further improve the outcome for ECMO-bridged patients, therefore, it has been proposed that non-invasive ventilation should be used rather than invasive mechanical ventilation. This could minimize the muscular deconditioning and ventilator-associated morbidity [42,47–49]. Furthermore, the possibility of keeping patients awake could avoid the hemodynamic consequences of general anesthesia and positive-pressure ventilation, especially in those with pulmonary hypertension. The study that enrolled patients bridged to transplant with ECMO as an alternative to invasive mechanical ventilation did in fact find significantly better six-month survival with ECMO than with mechanical ventilation (62% versus 35%) [26]; this suggests that preserving spontaneous breathing may keep patients in a better condition, with fewer of the drawbacks of mechanical ventilation.

Among the factors that can affect post-transplant outcome in patients bridged with ECMO, the most frequent are the duration of the bridge and the timing of the lung transplant [6]. Although patients can tolerate ECMO for long periods [34,50,51], any extra waiting time may significantly increase mortality [35]. Crotti *et al*. showed that patients who received a lung transplant after waiting more than 14 days had significantly higher rates of mortality and morbidity. ECMO-bridged patients should therefore be routinely re-assessed to make sure there are no exclusion criteria for lung transplantation, in order to optimize outcomes and avoid futile transplants [16].

The incidence of complications in ECMO patients awaiting lung transplant was similar to those previously reported in patients receiving ECMO for acute respiratory or cardiogenic shock [52]. It was not possible to compare the ICU and hospital lengths of stay for ECMO and non-ECMO patients because most of the studies gave no figures for the control group. Only two studies reported a shorter hospital stay for patients receiving awake-ECMO than for those given mechanical ventilation [26,35].

These observations suggest that ECMO might make it easier to optimize the clinical conditions of transplant candidates, mainly for more active participation in the activities of daily living, including ambulation, despite their critical respiratory illness. However, the length of stay depends on many factors, such as hospital mortality, which are not necessarily directly linked to support with ECMO or mechanical ventilation.

### Limitations

The present systematic review has several limitations. First, the studies included are not controlled or randomized trials but retrospective analyses of case series, with broad heterogeneity. Clearly, observational studies rarely provide sufficiently robust evidence to recommend changes to clinical practice or health policy decision-making. However, they are the only ones that provide any useful evidence for certain topics [31].

Second, the ventilatory strategy of the patients on ECMO bridging is not described (in terms of end-expiratory positive pressure, tidal volume, inspiratory pressure). Third, indications, type and duration of ECMO bridging differed among studies and some patients may have been described twice because they were reported in different studies [10,11,33,34,41]. Fourth, although we confined our literature search to the year 2000 onward because subsequent important advances were made in the technology for ECMO devices and mechanical ventilation recommendations that could have mixed up the results, three studies [11,34,36] enrolled patients before this period. Fifth, other extracorporeal life support strategies, potentially more easily manageable than ECMO, such as the artero-venous low flow extracorporeal carbon dioxide removal [53], the pulmonary artery-left atrium para-corporeal circuit configuration [54], and the minimally invasive low blood flow carbon dioxide removal systems [55], have been used as bridge to lung transplantation, but were excluded from this systematic review. The continuous technological advancement in the field of extracorporeal life support provides progressively more innovative devices, with the purpose to better suit specific patient populations. However, the notable difference of these newer strategies from ECMO in terms of management, complexity, and contribution to gas exchange, suggest they should be considered as a separate issue.

## Conclusions

Since its first application as a bridge to lung transplantation in patients with decompensating acute respiratory failure, ECMO support has gradually been used more frequently not only as salvage therapy, but also as a very promising alternative bridging strategy to mechanical ventilation, allowing more physiological respiratory assistance. However, given the quality and the wide heterogeneity among studies in this complex field, current clinical evidence does not permit any firm conclusions on the efficacy of ECMO as a bridge to lung transplantation in addition, or as an alternative to mechanical ventilation, and further prospective, more systematic multicenter trials are awaited. Future studies should ideally consider ECMO as part of a global algorithm of care for patients with end-stage lung disease, aiming at keeping them eligible for transplant despite refractoriness to maximal medical therapy, rather than just as an isolated means of respiratory support.

## Key messages

- Patients awaiting lung transplant are at high risk of death
- Mechanical ventilation can be required in end-stage severe acute respiratory failure
- ECMO support has been proposed as an alternative bridging strategy to mechanical ventilation
- ECMO support may provide acceptable one-year survival
- ECMO support should be part of a global algorithm of care for these patients

## Abbreviations

ARDS: acute respiratory distress syndrome
BOS: bronchiolitis obliterans syndrome
CA: cardiac arrest
CF: cystic fibrosis
CIP/CIM: critical illness polyneuropathy/critical illness myopathy
COPD: chronic obstructive pulmonary disease
CPB: cardiopulmonary by-pass
CWP: coal workers pneumoconiosis
DIC: disseminated intravascular coagulation
ECMO: extracorporeal membrane oxygenation
GI: gastrointestinal
H: hospital
ILD: interstitial lung disease
IP: interstitial pneumonia
LAS: lung allocation score
LOS: length of stay
LTx: lung transplant
MOF: multi-organ failure
MV: mechanical ventilation
PE: pulmonary embolism
PF: pulmonary fibrosis
PGD: primary graft dysfunction
PH: pulmonary hypertension
Post-LTx: post-lung transplantation
Pts: patients
PVOD: pulmonary veno-occlusive disease
Re-LTx: re-lung transplantation
Re-op: re-operation
SOFA: sequential organ failure assessment
UNOS: United Network of Organ Sharing
VA: veno-arterial
VV: veno-venous
NA: not available
